# Validation of the Comprehensive Feeding Practices Questionnaire among parents of 5- to 7-year-old children in Sweden

**DOI:** 10.3389/fpsyg.2023.1205427

**Published:** 2023-11-30

**Authors:** Zoë Morris, Åsa Norman, Liselotte Schäfer Elinder, Emma Patterson, Anna Warnqvist, Sara Raposo, Kristi Sidney Annerstedt

**Affiliations:** ^1^Department of Global Public Health, Karolinska Institutet, Solna, Sweden; ^2^Department of Clinical Neuroscience, Karolinska Institutet, Solna, Sweden; ^3^Centre for Epidemiology and Community Medicine (CES), Stockholm, Sweden; ^4^Swedish Food Agency, Uppsala, Sweden; ^5^Institute for Environmental Medicine, Karolinska Institutet, Solna, Sweden

**Keywords:** children, factor analysis, invariance, feeding practices, obesity, validation

## Abstract

**Introduction:**

Parents’ behaviours towards food and mealtimes, also known as parental feeding practices, are important in the development of children’s eating habits. The Comprehensive Feeding Practices Questionnaire (CFPQ) was designed to measure parental feeding practices. The aim of this study was to evaluate the validity of the CFPQ in Sweden and to assess how it performs across different groups of people.

**Methods:**

Data were from the baseline of a trial promoting children’s healthy dietary and physical activity behaviours, the Healthy School Start Plus intervention, conducted in 17 schools in the Stockholm region in Sweden. The CFPQ was completed by 263 parents (59% mothers) of 173 children, aged 5 to 7 years. Exploratory factor analysis and the omega reliability test were performed to identify the underlying factors in the data. Invariance testing was used to investigate the equivalence of these factors across parental sex, parental education and children’s weight status.

**Results:**

Five factors were identified: monitoring of children’s food intake, pressure to eat, restriction of food, use of food for emotional regulation, and healthy eating guidance. All five factors were invariant across parental sex and education, though some questions were excluded to achieve invariance. The monitoring, pressure to eat and emotional regulation factors were invariant across children’s weight status.

**Discussion:**

These results suggest that the CFPQ is valid for use in Sweden, amongst parents of children aged 5 to 7 years. The measurement invariance allows for comparisons of all five underlying factors across mothers and fathers and parental education levels, though across children’s weight status for only three factors. Due to the importance of parental feeding practices throughout childhood, this questionnaire should also be validated in other age groups in Sweden.

## 1 Introduction

Childhood obesity is a growing public health concern in Sweden, with 11.3% of 4-year-olds in Stockholm County being overweight or obese in 2021 (Region Stockholm, 2021). Being obese as a child significantly increases the risk of having a high Body Mass Index (BMI) later in life and is associated with premature mortality and an increased risk of developing many chronic diseases (Lindberg et al., 2020). Parents play a crucial role in their child’s eating habits and weight development through the physical, emotional and psychosocial environment they provide at home (Russell et al., 2018). Parental feeding practices (PFPs) are goal-oriented behaviours that parents use to influence when, what, and how much their children eat (Russell et al., 2018). Numerous studies have linked PFPs to a child’s weight status and many interventions have therefore targeted these behaviours (Shloim et al., 2015). Specific PFPs that have shown beneficial effects on child diet are modelling healthy eating behaviours and encouraging children to try fruit and vegetables (Shloim et al., 2015). Restrictive and controlling feeding practices, however, are frequently associated with a higher children’s BMI, whilst pressuring a child to eat more or all of the food on their plate has been associated with a lower BMI (Jansen et al., 2012). Giving or restricting food irrespective of hunger status has been linked to a higher children’s BMI (Jansen et al., 2012). This includes behaviours such as offering food if a child is sad to cheer them up or using food as a reward or punishment mechanism (Jansen et al., 2012). Certain parental characteristics, including low education level, have also been associated with higher risk of childhood obesity, a link which may, in part, be due to differences in their feeding practices (Nowicka et al., 2014).

Several instruments have been developed to measure PFPs, with one of the most used being the Comprehensive Feeding Practices Questionnaire (CFPQ). This was developed in 2007 in the United States and comprised of 49 questions, or items, organised into 12 factors, or constructs (Musher-Eizenman and Holub, 2007). The CFPQ has been validated in several countries but not yet in Sweden. The number of underlying, or latent, factors that have been identified in validation studies of the CFPQ vary, most commonly finding five to seven factors (Haszard et al., 2013; Saltzman et al., 2018; Arlinghaus et al., 2019; Minaie et al., 2019). Knowing which factors can be measured by a questionnaire allows it to be streamlined by removing questions irrelevant to the factors. This minimises the risk of participant fatigue when answering, thereby enhancing the quality of the data and increasing the number of responses (El-Den et al., 2020). Identifying the underlying factors also ensures that conclusions and comparisons are drawn on constructs which can reliably be measured by the questionnaire.

To be able to compare a construct (for example a PFP) between groups, such as parents of different sex or education levels, it is important that different groups of respondents interpret the questions similarly, and that the construct therefore has the same meaning in all groups. If this is not the case, differences in parents’ answers do not necessarily reflect true differences in feeding practices, but could be due to different interpretation of the questions (Cieciuch et al., 2019). This is investigated by measurement invariance testing which analyses participants’ answers to test whether they are interpreting questions in the same way. Only one paper has conducted measurement invariance testing on the CFPQ: a study using the CFPQ with parents of adolescents in Chile, which looked at measurement invariance across parental sex (del Valle et al., 2023).

The aim of the current study was to evaluate the validity of the CFPQ in the Swedish context, through conducting exploratory factor analysis and testing measurement invariance across parental sex, parental education level and across parents of children with different weight statuses. The resulting information on the factor structure and invariance of the CFPQ data will support future analyses on the role of PFPs on children’s eating habits and body weight development in Sweden.

## 2 Materials and methods

### 2.1 Study setting and population

Data were collected as part of the Healthy School Start Plus (HSSP) study, a cluster-randomised control trial, conducted in 17 schools in seven municipalities in the Stockholm region in Sweden, from 2017 to 2019 (Elinder et al., 2018). The HSSP intervention was designed to promote healthy eating habits and physical activity and, by doing so, prevent child obesity (Elinder et al., 2018). This intervention targeted the home environment by providing support, through school, to parents of 5- to 7-year-old children as they started school (Elinder et al., 2018). The HSSP programme had four components: a health information brochure for parents, motivational interviews for parents with the school nurse, classroom activities for children and homework to be completed together with parents, and an online self-test of type 2 diabetes risk for parents (Saaristo et al., 2005; Elinder et al., 2018). The purpose of the type 2 diabetes test was to inform parents of their risk of developing type 2 diabetes and consequently make them more likely to adapt their behaviour to reduce this risk (Elinder et al., 2018). Parents identified as high-risk for type 2 diabetes were also advised to contact their primary care provider for advice (Elinder et al., 2018).

The intervention targeted disadvantaged areas and therefore only schools where less than 50% of parents had a university education were eligible to participate in the HSSP (Elinder et al., 2018). Recruitment of participants for the HSSP trial was undertaken in three steps. Firstly, municipalities were recruited through convenience sampling where key persons in municipalities in mid-Sweden (e.g., head school nurses, educational boards, and public health practitioners) were contacted (Elinder et al., 2018). Municipalities interested in participating (*n* = 7) provided contact details for primary schools. Secondly, eligible schools were contacted and finally, in schools that agreed to participate (*n* = 17), parents were invited to participate in the study (Elinder et al., 2018). Parents were provided with written and oral information about the HSSP trial and signed written consents prior to the start of the trial. Both parents were invited to complete CFPQs though, in this study, only parents who responded to the CFPQ at baseline (August–October 2017) were included (*n* = 263). More detailed information on the recruitment process can be found in the HSSP study protocol (Elinder et al., 2018). The HSSP study received ethical approval from the Regional Ethical Review Board in Stockholm (No. 2017/711-31/1) (Elinder et al., 2018).

### 2.2 Measurements

The CFPQ used in the HSSP study was adapted from the original CFPQ (Musher-Eizenman and Holub, 2007), with 14 questions judged irrelevant for the Swedish context and removed. As an example, one of these was “*I often put my child on a diet to control his / her weight*.” The research team strongly anticipated that no parents (or very few) would admit to doing this in the Swedish cultural context. It was thought that keeping this question in would only add to participant burden by lengthening the questionnaire, and therefore it was removed. The questionnaire was translated into Swedish and back translated by two members of the research team, each of whom were native in one of the languages and highly proficient in the other (Elinder et al., 2018). In the translation process, items, or questions, were adapted to the Swedish context without losing any of their original meaning. Items were then discussed with researchers in the field of parenting and nutrition and finally pilot tested with four parents of 5- to 7-year-old children, who were not part of the HSSP trial, to test comprehensibility and cultural relevance of the translations (Elinder et al., 2018). The pilot participants were 36–47 years old, with a variety of educational backgrounds, an equal number of mothers and fathers of girls and boys and two of the parents were born in Sweden. The pilot testing resulted in minor changes to the wording of items (Elinder et al., 2018). The adapted version, consisting of 36 questions, was intended to map onto 10 behavioural factors from the original CFPQ (Musher-Eizenman and Holub, 2007): parental **monitoring** of food intake, use of food for **emotional regulation**, **encouraging** balanced and varied food intake, **pressure** to eat, **restriction** of food for **health** reasons, **restriction** of food for **weight** control, use of food as a **reward**, **involvement** of children in food planning and preparation, the home food **environment**, and parental **modelling** of food related behaviours (Elinder et al., 2018). Questions were answered using a five-point Likert scale, of either “*disagree, partly disagree, neutral, partly agree, completely agree*” for questions such as “*most of the food I have at home is healthy.*,” or “*never, rarely, sometimes, often, very often*” for questions such as “*do you give your child something to eat or drink when they are bored, even if you think that they are not hungry?*.” A full list of the questions and related response options are in the Supplementary material.

Parental education was self-reported and dichotomised into low (completed high school – maximum 12–13 years of education) and high (>12 years of education, including vocational training or a university degree) (Elinder et al., 2018). Parents also reported their country of birth, which was dichotomised into Nordic countries (Denmark, Finland, Iceland, Norway or Sweden) and non-Nordic countries. Children’s height and weight were measured by trained assistants, and BMI standard deviation scores (BMISDS) subsequently calculated (Elinder et al., 2018). Using the BMISDS, the International Obesity Task Force (IOTF) cut-offs were used to determine underweight, normal weight, overweight or obesity (Cole and Lobstein, 2012). Children in the overweight and obesity categories were combined into one group for this analysis (high BMI), and children in the normal and underweight categories were combined into one group (normal / low BMI).

### 2.3 Statistical analysis

Two CFPQ questions (Questions 11 and Q27 – Supplementary material) were negatively worded, and so their results were reverse coded to be comparable to the other questions. Parents with incomplete CFPQs were excluded. When a category on the five-point scale only had one or two people answering it, those answers were collapsed onto the next category to facilitate analysis. Such few answers do not provide ground for reliable analysis and collapsing was considered a more reasonable course of action than exclusion.

If Bartlett’s test of sphericity was significant (*p* ≤ 0.05) and the Kaiser-Meyer-Olkin (KMO) measure had a value ≥ 0.5, data were considered appropriate for factor analysis (Watkins, 2018).

#### 2.3.1 Exploratory factor analysis

The latent factors were identified using exploratory factor analysis (EFA) (Boateng et al., 2018). Horn’s parallel-analysis factor extraction method was used to identify the largest number of factors to test, and this result was corroborated by a scree plot (Watkins, 2018). The principal axis factor analysis, or common factor analysis, method was used for the factor extraction. Due to the ordinal nature of the data, polychoric correlations were used for this analysis, rather than Pearson’s correlations (Baglin, 2014; Bowen and Masa, 2015).

EFA was conducted using the number of factors identified by parallel-analysis factor extraction in the previous step. This analysis was performed using oblique rotation (Promax), the principal axis factoring method and polychoric correlations (Baglin, 2014; Watkins, 2018; Knekta et al., 2019). A pattern factor loading of ≥ |0.4| was considered acceptable (Haszard et al., 2013; Knekta et al., 2019). A solution had to meet four criteria to be accepted: (1) each factor must have at least three questions acceptably loaded onto it, (2) no questions should load onto more than one factor, (3) the internal reliability of all factors must be ≥ 0.7, (4) the factor must be theoretically reasonable (Watkins, 2018). To address the fourth criterion, the results were discussed within the research team to examine the theoretical basis to the relationships between the questions and the factors (Boateng et al., 2018). Internal reliability was measured using the omega reliability test (Hayes et al., 2020).

If an EFA solution was excluded, due to not fulfilling the above four criteria, EFA was performed again with one less factor specified than in the previous attempt (Watkins, 2018). This process continued until an acceptable solution was found. The questions in the final solution which did not load ≥ |0.4| onto any factors were considered for exclusion by the research team. If sufficient justification was made for including these questions, based on theoretical plausibility, they remained in the solution, mapped onto their highest loading factor (Knekta et al., 2019). As suggested by Knekta et al. (2019), EFA was then performed again, with only the included questions, to ensure that the same factor solution was found.

#### 2.3.2 Measurement invariance

Measurement invariance testing was used to investigate whether the latent factors were consistent across different subgroups of the study population (Putnick and Bornstein, 2016). Comparisons were made between mothers and fathers (also referred to as parental sex), between parents with a higher and lower education, and between parents of children with high BMI (overweight or obese) and normal / low BMI. If two parents of a child participated in the study, the child’s BMI was duplicated to be the same for both parents.

Due to the ordinal nature of the data, the estimation method used was the diagonally weighted least squares (DWLS) method (Li, 2016). Invariance testing was carried out by examining and comparing three models: a model with no constraints, a model where only factor loadings were constrained and a model where both factor loadings and intercepts were constrained (Putnick and Bornstein, 2016). These are referred to as “configural,” “metric” and “scalar” invariance models, respectively. The chi-square goodness of fit test, Comparative Fit Index (CFI), Tucker-Lewis Index (TLI) and Root Mean Square Error of Approximation (RMSEA) were used at each level as measures of model fit. DWLS is known to produce higher CFI and TLI values and lower RMSEA values than those produced by the maximum likelihood estimation method, which is commonly used in validation studies (Xia and Yang, 2019). The cut-off values for good invariance model fit were therefore: *p* > 0.05, CFI ≥ 0.98, TLI ≥ 0.98 and RMSEA ≤ 0.06 (Xia and Yang, 2019). A solution did not need to meet the threshold value for all four statistics to be considered a good model.

If a factor was noninvariant (did not meet the cut-offs for being an invariant model) at the configural level, individual questions, or items, were removed and the invariance testing re-run (Putnick and Bornstein, 2016). If removal of an item resulted in a significant improvement in the model fit, this item was considered for removal from the factor (Putnick and Bornstein, 2016). If a factor was invariant at the configural level, but noninvariant at the metric or scalar level, partial invariance testing was performed (Putnick and Bornstein, 2016). Partial invariance testing involves releasing the constraints for one or more of the items and re-running the tests (Putnick and Bornstein, 2016). If partial metric or scalar measurement invariance was achieved, with at least two items being invariant (not released in the partial invariance testing), the factor was classified as invariant at that level (Cieciuch et al., 2019). Once a factor was found to be invariant at all levels (configural, metric and scalar models), these models were compared to determine whether the factor was invariant overall (Putnick and Bornstein, 2016). Each model was compared to the model with one more constraint than it (configural was compared to metric, and metric was compared to scalar) (Putnick and Bornstein, 2016). Two tests of the differences between the models were computed: chi-squared test, and difference in CFI values (CFI difference was calculated by subtracting the CFI value of the less constrained model from the CFI value of the more constrained model) (Putnick and Bornstein, 2016). To be considered invariant, a chi-square value of *p* ≥ 0.05 and / or a difference in CFI ≤ −0.01 was needed (Putnick and Bornstein, 2016).

Stata (version 16.1) (StataCorp, 2019) and R (version 4.1.2) (R Core Team, 2021) [including three R packages: *Lavaan* (Rosseel, 2012)*, psych* (Revelle, 2022) and *EFAtools* (Steiner and Grieder, 2019)] were used for these analyses.

## 3 Results

### 3.1 Study population

This study included 263 parents (154 mothers, 109 fathers) with 173 individual children (Table 1). Amongst parents who provided demographic information (*n* = 259), 59% of parents had a high level of education and 68% of parents were born in a Nordic country (97% of whom were born in Sweden) with the remaining 32% of parents being born in 36 different countries.

**Table 1:** Descriptive characteristics of the study population.

Characteristic	Category	*n* (%)
Parent (*n* = 263)	Mother	154 (58.6)
	Father	109 (41.4)
Parental education (*n* = 259)^a^	Low	107 (41.3)
	High	152 (58.7)
Parental region of birth (*n* = 259)^b^	Nordic	177 (68.3)
	Non-Nordic	82 (31.7)
Child BMI (*n* = 173)^c^	Underweight	11 (6.4)
	Normal weight	120 (69.4)
	Overweight	23 (13.3)
	Obese	16 (9.2)
	Data missing	3 (1.7)

BMI, Body Mass Index.

a Low education = maximum completed high school, High education = university / vocational training after high school.

b Nordic = Denmark, Finland, Iceland, Norway and Sweden.

c International Obesity Task Force categories.

Ten CFPQ response values were collapsed onto the closest category, for reasons explained previously, in Section 2.3.

Bartlett’s test of sphericity was significant (*p* < 0.05) and the KMO was 0.78, indicating that the data were suitable for factor analysis.

### 3.2 Exploratory factor analysis

Parallel-analysis factor extraction suggested a seven-factor solution. A first EFA was therefore carried out using seven factors, however this solution did not fulfil the first criterion for acceptance; one factor only had two questions loaded onto it. The six-factor solution was rejected for the same reason. The five-factor solution fulfilled all four criteria and was accepted (Table 2). Questions 17 and 21 were included in the solution, despite loading < 0.4, as they were considered theoretically relevant by the research team. The omega test for internal reliability was ≥ 0.7 for all factors (Table 2). Eight questions were not included in this solution (Questions 10, 11, 14, 18, 24, 26, 27, and 31).

**Table 2:** Results from the exploratory factor analysis.

Questions (Q) by factor	Questions	Factor loading	Original CFPQ subscale
**Monitoring (ω** ^a^ **= 0.970)**			
Q1	How much do you keep track of / are aware of how much and how often your child eats sweet things?	0.95	Monitoring
Q2	How much do you keep track of / are aware of how much and how often your child eats snacks?	0.95	Monitoring
Q3	How much do you keep track of / are aware of how much and how often your child eats fatty / fried food?	0.95	Monitoring
Q4	How much do you keep track of / are aware of how much and how often your child drinks sweet drinks?	0.95	Monitoring
**Pressure to eat (ω = 0.778)**			
Q12	My child should always eat all of the food on their plate.	0.44	Pressure
Q23	If my child says “I am not hungry,” I try to get them to eat anyway.	0.79	Pressure
Q29	If my child only eats a small portion, I try to get them to eat more.	0.84	Pressure
Q36	When my child says that they have finished eating, I try to get them to eat one / a few more bites.	0.78	Pressure
**Restriction (ω = 0.857)**			
Q15	If I did not regulate my child’s eating, they would eat too much of their favourite dishes.	0.43	Restriction for health reasons
Q20	I encourage my child to eat less so they will not get fat.	0.88	Restriction for weight control
Q21	If I did not regulate my child’s eating, they would eat too much junk food / unhealthy food.	0.37	Restriction for health reasons
Q22	I give my child small portions at mealtimes to make sure that they do not gain weight.	0.87	Restriction for weight control
Q25	If my child eats more than usual at one mealtime, I try to limit how much they eat at the next mealtime.	0.75	Restriction for weight control
Q30	I have to be sure that my child does not eat too much of their favourite food.	0.68	Restriction for health reasons
**Emotional regulation (ω = 0.817)**			
Q5	When your child is fussy, is giving them something to eat or drink the first thing you do?	0.65	Emotional regulation
Q6	Do you give your child something to eat or drink when they are bored, even if you think that they are not hungry?	0.79	Emotional regulation
Q7	Do you give your child something to eat or drink when they are upset, even if you think that they are not hungry?	0.85	Emotional regulation
Q13	I offer my child their favourite food in exchange for good behaviour.	0.48	Food as reward
Q17	I offer sweet things (e.g., candy, ice cream, cookies) to my child as a reward when they have been good.	0.33	Food as reward
**Healthy eating guidance (ω = 0.891)**			
Q8	Do you encourage your child to eat healthy food before they eat unhealthy food?	0.59	Encouraging balance and variety
Q9	Most of the food I keep in the house is healthy.	0.63	Environment
Q16	There are several different kinds of healthy food for my child to choose between at every mealtime at home.	0.40	Environment
Q19	I tell my child that healthy food tastes good.	0.68	Encouraging balance and variety
Q28	I encourage my child to eat many different kinds of food.	0.45	Encouraging balance and variety
Q32	I show my child what healthy eating is by eating healthily myself.	0.78	Modelling
Q33	I try to eat healthy food in front of my child even if it is not my favourite food.	0.68	Modelling
Q34	I try to show enthusiasm about eating healthy food.	0.84	Modelling
Q35	I show my child how much I enjoy eating healthy food.	0.83	Modelling

a ω = Omega. A measure of the reliability of the factor.

The name of the factor is shown in bold, with its contributing questions listed underneath. For a full list of questions, including excluded questions, see the Supplementary material. The final column details the subscale that the question was part of in the original Comprehensive Feeding Practices Questionnaire (CFPQ) (Musher-Eizenman and Holub, 2007).

The five factors included in the solution were: **monitoring** of food intake, **pressure** to eat, **restriction** of food, use of food for **emotional regulation**, and **healthy eating guidance** (Table 2). The restriction factor found in this analysis was a combination of three questions from the original “restriction for weight control” subscale and three questions from the “restriction for health reasons” subscale. The new emotional regulation factor included all three questions from the original “emotional regulation” subscale, and two of the three questions from the original “food as reward” subscale. The healthy eating guidance factor was not an original CFPQ subscale but included a mixture of items from different original subscales, including environment, modelling, and encouraging balance and variety.

### 3.3 Measurement invariance

Measurement invariance testing results (configural, metric and scalar) by group (parental sex, education level and child weight status) are shown in Table 3. The questions that contributed to the final invariance results are listed after the respective factor name.

**Table 3:** Results from the invariance testing for the five-factor exploratory factor analysis solution (monitoring, pressure to eat, restriction, emotional regulation and healthy eating guidance) for three group comparisons (parental sex, parental education level and child weight status).

Model	χ^2^ (df)^a^	CFI	TLI	RMSEA	Δχ^2^ *p*-value	ΔCFI	Model acceptance
**Parental sex**							
Mother *n* = 154, Father *n* = 109							
*Monitoring* – Questions 1,2,3,4							
Configural	18.765 (4)^*^	0.999	0.998	0.168			Accepted
Metric	17.632 (7)	0.999	0.999	0.108	0.676	0	Accepted
Scalar	23.973 (15)	1	1	0.068	0.904	0.001	Accepted
*Pressure to eat* – Questions 12,23,29,36							
Configural	7.728 (4)	0.996	0.987	0.085			Accepted
Metric	11.835 (7)	0.994	0.990	0.073	0.156	−0.002	Accepted
Scalar	22.536 (18)	0.995	0.996	0.044	0.534	0.001	Accepted
*Restriction* – Questions 15,20,22,25,30 (removed 21)							
Configural	14.492 (10)	0.996	0.991	0.059			Accepted
Metric	18.014 (14)	0.996	0.994	0.047	0.366	0	Accepted
Scalar	36.284 (28)	0.992	0.994	0.048	0.144	−0.004	Accepted
*Emotional regulation* – Questions 5,6,7,13,17							
Configural	22.403 (10)	0.985	0.970	0.097			Accepted
Metric	28.486 (14)	0.983	0.975	0.089	0.122	−0.002	Accepted
Scalar	27.931 (24)	0.995	0.996	0.035	1	0.012	Accepted
*Healthy eating guidance* – Questions 8,9,16,19,33,34,35 (removed 28 and 32)							
Configural	49.545 (28)^*^	0.991	0.987	0.077			Accepted
Metric	74.101 (34)^*^	0.984	0.980	0.095	0.003	−0.007	Accepted
Scalar	65.259 (53)	0.995	0.996	0.042	1	0.011	Accepted
**Parental education** ^b^							
Low *n* = 107, High *n* = 152							
*Monitoring* – Questions 1,2,3,4							
Configural	20.536 (4)^*^	0.999	0.998	0.179			Accepted
Metric	27.012 (7)^*^	0.999	0.999	0.149	0.012	0	Accepted
Scalar	30.505 (14)^*^	0.999	1	0.096	1	0	Accepted
*Pressure to eat* – Questions 12,23,29,36							
Configural	10.911 (4)	0.992	0.975	0.116			Accepted
Metric	18.436 (7)	0.986	0.977	0.113	0.034	−0.006	Accepted
Scalar	30.835 (18)	0.985	0.990	0.074	0.326	−0.001	Accepted
*Restriction* – Questions 15,20,22,25,30 (removed 21)							
Configural	7.912 (10)	1	1	0			Accepted
Metric	14.818 (14)	0.999	0.999	0.021	0.254	−0.001	Accepted
Scalar	26.283 (28)	1	1	0	0.583	0.001	Accepted
*Emotional regulation* – Questions 5,6,7,13,17							
Configural	9.877 (10)	1	1	0			Accepted
Metric	55.007 (14)^*^	0.952	0.932	0.151			Rejected
Partial metric	1.272 (2)	1	1	0	<0.01	0	Accepted
Scalar	62.419 (24)^*^	0.955	0.963	0.112			Rejected
Partial scalar	5.169 (7)	1	1	0	0.607	0	Accepted
*Healthy eating guidance* – Questions 8,9,16,19,33,34,35 (removed 28 and 32)							
Configural	53.609 (28)^*^	0.989	0.984	0.084			Accepted
Metric	62.290 (34)^*^	0.988	0.985	0.080	0.072	−0.001	Accepted
Scalar	82.496 (53)^*^	0.987	0.990	0.066	0.562	−0.001	Accepted
**Child BMI status** ^c^							
Underweight / normal *n* = 199, Overweight / obesity *n* = 59							
*Monitoring* – Questions 1,2,3,4							
Configural	21.954 (4)^*^	0.999	0.997	0.187			Accepted
Metric	20.195 (7)^*^	0.999	0.999	0.121	0.142	0	Accepted
Scalar	25.806 (15)	0.999	1	0.075	0.989	0	Accepted
*Pressure to eat* – Not possible – unequal categories							
*Restriction* – Questions 20,21,22,25,30 (removed 15)							
Configural	21.515 (10)	0.985	0.971	0.095			Accepted
Metric	20.999 (14)	0.991	0.987	0.062	0.399	0.006	Accepted
Scalar	42.971 (28)	0.981	0.987	0.065	0.111	−0.010	Accepted
*Emotional regulation* – Questions 5,6,7,13,17							
Configural	14.189 (10)	0.994	0.988	0.057			Accepted
Metric	23.240 (14)	0.987	0.982	0.072	0.160	−0.007	Accepted
Scalar	34.180 (22)	0.983	0.985	0.066	0.197	−0.004	Accepted
*Healthy eating guidance* – Not possible – unequal categories							

The questions that contributed to the displayed invariance results are listed after the factor name. CFI, Comparative Factor Index; df, degrees of freedom; RMSEA, Root Mean Square Error of Approximation; TLI, Tucker-Lewis Index; χ^2^, chi-squared. *p < 0.01.

a The robust estimates are shown here.

b Low education = maximum completed high school, High education = university / vocational training after high school.

c International Obesity Task Force categories.

All five factors were invariant across parental sex and parental education, though three questions needed to be removed, from both group comparisons, for invariance to be achieved: Question 21 from the restriction factor and Questions 28 and 32 from the healthy eating guidance factor. Partial metric and scalar measurement invariance were obtained for the emotional regulation factor for parental education.

Only three comparisons could be made for parents of children with different weight statuses due to the two weight status groups having different ranges and therefore unequal categories in the pressure to eat and healthy eating guidance factors. The monitoring and emotional regulation factors were accepted as invariant, as was the restriction factor, once Question 15 had been removed.

## 4 Discussion

The aim of this study was to evaluate the validity of the CFPQ in a sample of parents of 5- to 7-year-old children in Sweden. Similar to other CFPQ validation studies (Table 4), this factor analysis could not reproduce the original CFPQ’s 12-factor structure. In this study population, five PFP factors were found: monitoring of food intake, pressure to eat, restriction of food, emotional regulation, and healthy eating guidance. Of the 36 CFPQ questions used in the HSSP trial, eight questions were found to be extraneous to these five factors. All five factors were invariant across parental sex and education. Monitoring of food intake, restriction and emotional regulation were invariant across children’s weight status.

**Table 4:** Papers using exploratory factor analysis to assess the validity of the Comprehensive Feeding Practices Questionnaire.

**Lead author/s**	Musher-Eizenman and Holub (2007)	Melbye et al. (2011)	Haszard et al. (2013)	Mais et al. (2015)	Warkentin et al. (2016)	Al-Qerem et al. (2017)	Saltzman et al. (2018)	Arlinghaus et al. (2019)	Minaie et al. (2019)	Rahmaty et al. (2022)	del Valle et al. (2023)	Morris (Current study)
**Year**	2007 (3^rd^ study)	2011	2013	2015	2016	2017	2018	2019	2019	2022	2023	2023
**Country**	United States	Norway	New Zealand	Brazil	Brazil	Jordan	United States	United States	Iran	United States	Chile	Sweden
**No. factors**	12	10	5	6	6	11	5	5	7	13^a^	4	5
**Factor names**	Monitoring Pressure RH RW Emotional regulation Food as reward Child control Teaching nutrition Environment Modelling Encouraging balance Involvement	Monitoring Pressure RH RW Child control Teaching nutrition Environment Modelling Encouraging balance Involvement	Monitoring Pressure Restriction Child control HEG	Monitoring Pressure RH RW Emotional regulation / food as reward HEG	Monitoring Pressure RH RW Emotional regulation / food as reward HEG	Monitoring Pressure RH RW Emotional regulation Food as reward Child control Teach and encourage Environment Modelling Involvement	Monitoring Pressure RH RW HEG	Monitoring RW HEG Promotion of over-consumption Healthy eating variety	Monitoring Pressure Restriction Child control Emotional regulation Modelling HEG	Monitoring Pressure RH Emotional regulation Food as reward Child control Teaching nutrition and balance Environment Modelling Involvement Limiting amount Limiting type* No snacks / sweets in house*	Monitoring Child control RW Modelling	Monitoring Pressure Restriction Emotional regulation HEG
**Child age**	1–8 years (4.3 mean)	10–12 years	6.5 years (mean)	5–9 years	2–5 years	6–12 years (9.1 mean)	4.8 years (mean)	3–5 years	2–5 years	3–5 years (4 mean)	12–16 years (12.5 mean)	5–7 years
**Sample size**	152 mothers	963 parents	1,013 children	659 children	402 children	970 children	260 children	187 children	300 children	437 caregivers	946 parents	263 parents
**Mothers / fathers**	Mothers	85% mothers 12% fathers	51% mothers 49% fathers	46% mothers 54% fathers	93% mothers 7% fathers	Mothers	Mothers	Mothers	Mothers	90% women 10% men	50% mothers 50% fathers	59% mothers 41% fathers

Papers were found using a literature search of PubMed with the search terms (“Comprehensive Feeding Practices Questionnaire”) AND (validation OR factor analysis). Twenty-three papers were identified which was reduced to ten after applying the following inclusion selection criteria: (1) papers validating the Comprehensive Feeding Practices Questionnaire (CFPQ), (2) parents completing the CFPQ about their children, (3) not completing the CFPQ retrospectively, (4) not conducting only confirmatory factor analysis. Musher-Eizenman and Holub conducted confirmatory factor analysis, but this paper is included in the table as it describes the development of the original CFPQ. HEG, Healthy eating guidance; RH, Restriction for health, RW, Restriction for weight.

a Two of these factors (*) had fewer than three loading questions, a criterion for factor acceptance in the other papers, therefore this result cannot be directly compared.

This five-factor solution is consistent with other validation studies (Haszard et al., 2013; Mais et al., 2015; Warkentin et al., 2016; Saltzman et al., 2018; Arlinghaus et al., 2019), which almost always identify the monitoring and pressure to eat factors (Table 4). Both of these factors retained the same questions as the original CFPQ factors (Musher-Eizenman and Holub, 2007), a finding consistent across the majority of CFPQ validation studies (Haszard et al., 2013; Warkentin et al., 2016; Al-Qerem et al., 2017; Arlinghaus et al., 2019; Minaie et al., 2019). These studies have commonly found high factor loadings for the monitoring questions, as found in this analysis, perhaps due to the similar nature of the questions. The questions differ only in the food groups being inquired about (sweet food, sugary drinks, snacks, fatty foods), but these are deemed important to ask about separately.

The restriction factor included a mixture of questions previously in the “restriction for weight control” and “restriction for health reasons” subscales. This combination has also been found in previous validation studies (Haszard et al., 2013; Minaie et al., 2019), with the original CFPQ paper acknowledging that parents may have difficulty identifying their motivation for restricting food (Musher-Eizenman and Holub, 2007). Validation studies which retain them as two separate factors (Melbye et al., 2011; Mais et al., 2015; Warkentin et al., 2016; Al-Qerem et al., 2017; Saltzman et al., 2018) often find strong correlations between the factors.

The emotional regulation factor was a mixture of the original emotional regulation subscale questions and two questions from the food as reward subscale, a combination which has been found in previous validation studies (Mais et al., 2015; Warkentin et al., 2016). Reward involves giving or restricting food depending on an action which is associated with an emotion, be it happiness for an accomplishment or sadness from being badly behaved. It was therefore considered reasonable that these reward questions were combined with the emotional regulation factor.

Healthy eating guidance was not one of the 12 factors in the original CFPQ (Musher-Eizenman and Holub, 2007) but has been identified as a factor in other CFPQ validation studies (Haszard et al., 2013; Mais et al., 2015; Warkentin et al., 2016; Saltzman et al., 2018; Arlinghaus et al., 2019; Minaie et al., 2019). It includes questions from three original CFPQ subscales: environment, modelling, and encouraging balance and variety. Studies assessing CFPQ results have found high correlations between these three subscales (Haszard et al., 2013). In this analysis, only the two environmental questions regarding the presence of healthy food in the house (Questions 9 and 16) load onto the factor, not the questions about the presence of unhealthy food (Questions 11 and 27), which supports the nature of the factor as an overall health promoting behaviour, rather than a restrictive behaviour.

Whilst it may be expected that the results from this study would be similar to a validation study conducted in another Nordic country, a Norwegian study identified 10 factors (Melbye et al., 2011). One explanation for this difference may be that their cohort of children was older (10–12 years) than in this study (5–7 years), and PFPs change depending on the age of the child (Shloim et al., 2015). However, amongst the papers which conduct EFA on the CFPQ (Table 4), there is not an obvious association between the age of the children and the number of factors identified. The Norwegian study did have a considerably larger sample size than this study, yet a much smaller proportion of fathers. These aforementioned study characteristics may have an effect on the factors identified, but further analysis is needed to establish this.

Only one study has investigated the measurement invariance of the CFPQ (del Valle et al., 2023), despite invariance being essential for using the scale to compare different groups of participants. Their study, however, is using parents of adolescents and is conducted in Chile, therefore their results may not be comparable in our age group and in Sweden. Our invariance testing is therefore unique but urgently should be done in all other countries using the CFPQ, before important conclusions are drawn based on potentially noninvariant results. Our invariance results indicate that all five factors are invariant across parental sex and parental education levels. Data from the HSSP trial can therefore be compared between these groups. It is common for studies to only include mothers (Table 4), and consequently much less research has been conducted regarding fathers’ feeding practices. This is an important area for research, especially in the Nordic context where it is common for fathers to take on a large responsibility for childcare. Previous studies have found associations between parental education and feeding practices (Nowicka et al., 2014); the invariance of the factors across parental education level enables this to be investigated in the HSSP data.

The questions which were found to be noninvariant (Questions 15, 21, 28, and 32) should not be included when comparing factors between the groups in which they were noninvariant. They would not, however, need to be removed from the Swedish questionnaire. The noninvariance of these questions may mean that the groups are interpreting the question differently or the question or concept may have different meanings to the different groups (Putnick and Bornstein, 2016). Useful information on the differences between groups, or insights into the questions themselves, can be gained from investigating this noninvariance (Hammack-Brown et al., 2022). It is particularly interesting that the same three questions (Questions 21, 28, and 32) were noninvariant for both parental sex and parental education. These three questions do not have obvious connections; they are not worded similarly and are enquiring about different behaviours. Questions 15 and 21 are, however, similar questions. They both begin with “If I did not regulate my child’s eating….” Question 15 then asks whether the child *would* eat too much of their favourite food, whilst Question 21 asks whether the child *would* eat too much junk food. These are both speculative questions, unlike all of the other questions which enquire about the practices that the parent currently undertakes. It is possible that these could be interpreted differently; some parents may interpret it as asking whether they currently have to regulate this on a daily basis, whereas some may interpret it as, if their child was theoretically given as much of this food as they like, would they need to regulate how much the child eats, or would they stop eating on their own? One reason for the noninvariance of Question 28 (“I encourage my child to eat many different kinds of food.”) could be different interpretations of whether this is referring to all kinds of food or different kinds of healthy food, especially as the other questions are clear in whether they are referring to healthy or unhealthy food. No explanation could be found for the noninvariance of Question 32. Multilevel structural equation modelling can explain sources of and reasons for noninvariance and would therefore be valuable to conduct on all of the noninvariant questions (Cieciuch et al., 2019).

### 4.1 Strengths and limitations

One strength of this study was that the data were consistently treated as ordinal. It is common practice to treat Likert scale data from the CFPQ as continuous, which can lead to inaccurate results and underestimation of factor loadings (Li, 2016). Our use of techniques appropriate for ordinal data, such as using polychoric correlations and diagonally weighted least squares (DWLS) estimation methods, reduced this risk. Whilst DWLS was the suitable estimation method for ordinal data, it has been shown to be sensitive to small sample sizes and non-normal data (Distefano and Morgan, 2014). For this reason, an additional analysis to confirm the invariance findings should be performed when a larger dataset is available.

The heterogenous nature of the data was another strength of this study. Forty-one percent of parents had a low education level which, whilst still fewer than the Swedish average [53% of those aged 25–64 years in 2020 (Westling, 2021)], is more representative of the population than in many studies with large proportions of highly educated parents. This supports the external validity of the questionnaire to Sweden as a whole but may hinder the generalisability in areas of higher socioeconomic status. The large number of fathers was another strength which increases the external validity of the questionnaire.

Whilst the sample size was not small, it was not large enough to conduct all desired invariance tests. For the children’s BMI invariance tests there were significantly fewer parents in the high children’s BMI group. This meant that the range of that group’s answers was much smaller than for the lower BMI group which made it impossible to perform some invariance tests.

### 4.2 Future directions and conclusions

Further studies on the validity of the CFPQ are required to assess the generalisability of these findings. The external validity of the CFPQ could be affected by other factors such as a child’s age, additional indicators of parent’s socioeconomic status and parent’s country of birth. Validating the CFPQ with children of other ages and conducting measurement invariance tests for additional parental sociodemographic factors would be useful. Future invariance testing with a larger sample size would also allow for invariance across child’s weight status to be evaluated for all factors.

In conclusion, the CFPQ has high validity for use in the HSSP study population. Five PFPs can be evaluated in this population, and removal of the non-loading questions could streamline the CFPQ. The five feeding practice factors identified can be reliably compared between mothers and fathers and across different levels of parental education. The CFPQ can therefore be used in Sweden when investigating the importance of PFPs for children’s dietary habits and body weight development.

## Data availability statement

The data analysed in this study are subject to the following licenses/restrictions: the datasets generated and/or analysed are not publicly available due to ethical reasons but are available from the corresponding author on reasonable request. A data sharing agreement will have to be signed. Requests to access these datasets should be directed to KS, kristi.sidney@ki.se.

## Ethics statement

The HSSP study received ethical approval from the Regional Ethical Review Board in Stockholm (No. 2017/711-31/1). Parents or legal guardians provided written informed consent for their own and their child’s participation in the HSSP study.

## Author contributions

ZM, ÅN, LE, and KS conceptualised the study. ÅN, EP, and LE collected the data. ZM and KS performed the analyses. AW provided statistical support. ZM drafted the manuscript. All authors contributed to the article and approved the submitted version.
