# Supplementary material for: Validation of the Comprehensive Feeding Practices Questionnaire among parents of 5- to 7-year-old children in Sweden
Source: Front Psychol. 2023 Nov 30;14:1205427. doi: 10.3389/fpsyg.2023.1205427 (PMC10719845; doi:10.3389/fpsyg.2023.1205427)
Supplement: Supplementary file 1 [file Table_1.DOCX]

Supplementary Material

**Questions from the Comprehensive Feeding Practices Questionnaire used in the Healthy School Start Plus Study.**

These questions are back translated from the Swedish questions used in the Healthy School Start Plus study. The questions in Swedish are available on request.

Questions with answers “*never, rarely, sometimes, often, very often*”:

1. How much do you keep track of / are aware of how much and how often your child eats sweet things (e.g., sweets, ice cream, cookies)?
2. How much do you keep track of / are aware of how much and how often your child eats snacks (e.g., chips, cheese puffs, salted crackers)?
3. How much do you keep track of / are aware of how much and how often your child eats fatty / fried food (e.g., fries, pizza)?
4. How much do you keep track of / are aware of how much and how often your child drinks sweet drinks (soda, juice, O’boy)?
5. When your child is fussy, is giving them something to eat or drink the first thing you do?
6. Do you give your child something to eat or drink when they are bored, even if you think that they are not hungry?
7. Do you give your child something to eat or drink when they are sad, even if you think that they are not hungry?
8. Do you encourage your child to eat healthy food before they eat unhealthy food?

Questions with answers “*disagree, partly disagree, neutral, partly agree, completely agree*”:

1. Most of the food I keep in the house is healthy.
2. I involve my child in the planning of the family meals.
3. I usually have many snacks (e.g., chips, cheese puffs, salted crackers) at home.
4. My child should always eat all of the food on their plate.
5. I offer my child their favourite food in exchange for good behaviour.
6. I let my child help prepare family meals.
7. If I did not regulate my child’s eating, they would eat too much of their favourite dishes.
8. There are several different kinds of healthy food for my child to choose between at every mealtime at home.
9. I offer sweet things (e.g., sweets, ice cream, cookies) to my child as a reward when they have been good.
10. I encourage my child to try new foods.
11. I tell my child that healthy food tastes good.
12. I encourage my child to eat less so they will not get fat.
13. If I did not regulate my child’s eating, they would eat too much junk food / unhealthy food.
14. I give my child small portions at mealtimes to make sure they do not gain weight.
15. If my child says “I am not hungry”, I try to get them to eat anyway.
16. I encourage my child to participate in grocery shopping for the family.
17. If my child eats more than usual at one mealtime, I try to limit how much they eat at the next mealtime.
18. I do not let my child have sweet things (e.g., sweets, ice cream, cookies) or dessert if they have misbehaved.
19. I usually have many sweet things (e.g., sweets, ice cream, cookies) at home.
20. I encourage my child to eat many different kinds of food.
21. If my child only eats a small portion, I try to get them to eat more.
22. I have to be sure that my child does not eat too much of their favourite food.
23. I have to be sure that my child does not eat too many sweet things (e.g., sweets, ice cream, cookies).
24. I show my child what healthy eating is by eating healthily myself.
25. I try to eat healthy food in front of my child, even if it is not my favourite food.
26. I try to show enthusiasm about eating healthy food.
27. I show my child how much I enjoy eating healthy food.
28. When my child says that they have finished eating, I try to get them to eat one / a few more bites.
